# Conservative versus tailored surgical treatment in patients with first time lateral patella dislocation: a randomized-controlled trial

**DOI:** 10.1186/s13018-021-02513-3

**Published:** 2021-06-13

**Authors:** M. Liebensteiner, A. Keiler, R. El Attal, P. Balcarek, F. Dirisamer, J. Giesinger, G. Seitlinger, M. Nelitz, A. Keshmiri, J. Frings, Ch. Becher, P. Kappel, D. Wagner, G. Pagenstert

**Affiliations:** 1 Department of Orthopaedics and Traumatology, Medical University of Innsbruck, Anichstr. 35, 6020 Innsbruck, Austria; 2 Landeskrankenhaus Feldkirch, Carinagasse 41, 6800 Feldkirch, Austria; 3 ARCUS Sportklinik, Rastatter Str. 17-19, 75179 Pforzheim, Germany; 4 Orthopädie & Sportchirurgie, Karl-Leitl-Str. 1, 4048 Linz, Puchenau Austria; 5 OSMI — Research Unit for Orthopaedic Sports Medicine and Injury Prevention, UMIT — Private University for Health Sciences, Medical Informatics and Technology, Eduard-Wallnöfer-Zentrum 1, 6060 Hall in Tirol, Austria; 6 University Hospital of Psychiatry II, Medical University of Innsbruck, Anichstr. 35, 6020 Innsbruck, Austria; 7 Orthofocus, Guggenbichlerstr. 20, 5026 Salzburg, Austria; 8 MVZ Oberstdorf, Kliniken Kempten/Oberallgäu, Trettachstr. 16, 87561 Oberstdorf, Germany; 9 MVZ im Helios, Helene-Weber-Allee 19, 80637 Munich, Germany; 10 Department of Trauma and Orthopaedic Surgery, University Medical Center Hamburg-Eppendorf, Martinistr. 52, 20246 Hamburg, Germany; 11 International Center for Orthopaedics, ATOS Clinic, Bismarckstr. 9-15, 69115 Heidelberg, Germany; 12 Department of Orthopaedics, Trauma Surgery and Sports Medicine, Cologne Merheim Medical Center, University of Witten/Herdecke, Ostmerheimer Str. 200, 51109 Cologne, Germany; 13 Hessingpark-Clinic GmbH, Hessingstr. 17, 86199 Augsburg, Germany; 14 CLARAHOF Orthopaedics, Merian-Iselin-Hospital — Swiss Olympic Medical Center, Clarahofweg 19a, 4058 Basel, Switzerland

**Keywords:** Patella, Instability, Dislocation, Conservative treatment, Tailored surgical treatment

## Abstract

**Background:**

Patellar instability has a high incidence and occurs particularly in young and female patients. If the patella dislocates for the first time, treatment is usually conservative. However, this cautious approach carries the risk of recurrence and of secondary pathologies such as osteochondral fractures. Moreover, there is also risk of continuous symptoms apparent, as recurrent patella dislocation is related to patellofemoral osteoarthritis as well. An initial surgical treatment could possibly avoid these consequences of recurrent patella dislocation.

**Methods:**

A prospective, randomized-controlled trial design is applied. Patients with unilateral first-time patella dislocation will be considered for participation. Study participants will be randomized to either conservative treatment or to a tailored patella stabilizing treatment. In the conservative group, patients will use a knee brace and will be prescribed outpatient physical therapy. The surgical treatment will be performed in a tailored manner, addressing the pathologic anatomy that predisposes to patella dislocation.

The Banff Patellofemoral Instability-Instrument 2.0, recurrence rate, apprehension test, joint degeneration, and the Patella Instability Severity Score will serve as outcome parameters. The main analysis will focus on the difference in change of the scores between the two groups within a 2-year follow-up.

Statistical analysis will use linear mixed models. Power analysis was done for the comparison of the two study arms at 2-year follow-up with regard to the BPII Score. A sample size of N = 64 per study arm (128 overall) provides 80% power (alpha = 0.05, two-tailed) to detect a difference of 0.5 standard deviations in a t-test for independent samples.

**Discussion:**

Although several studies have already dealt with this issue, there is still no consensus on the ideal treatment concept for primary patellar dislocation. Moreover, most of these studies show a unified surgical group, which means that all patients were treated with the same surgical procedure. This is regarded as a major limitation as surgical treatment of patella dislocation should depend on the patient’s anatomic pathologies leading to patellar instability. To our knowledge, this is the first study investigating whether patients with primary patella dislocation are better treated conservatively or operatively with tailored surgery to stabilize the patella.

**Trial registration:**

The study will be prospectively registered in the publicly accessible database www.ClinicalTrials.gov.

## Introduction

Instability of the patella has a high incidence, particularly in the young and female population. Because the vast majority of unstable patella are unstable towards *lateral* and because instability is objective when the patella is fully *dislocated* the term “lateral patella dislocation (LPD)” is used throughout this study protocol.

*First time* or *primary* LPD is often treated conservatively. Although this is a cautious approach, it bears risks of recurrence and secondary pathologies like osteochondral fractures or patellofemoral osteoarthritis [1, 2]. Therefore, it might well be speculated whether primary LPD should better be treated surgically, not only for short time clinical improvement, but also for prevention of PF osteoarthritis. With this regard, several researchers already dealt with that issue. Table 1 gives an overview on current evidence on conservative vs. surgical treatment of patients with first-time lateral patella dislocation. However, only studies with level of evidence 1 or 2 were taken into account.

**Table 1:** Current evidence on conservative vs. surgical treatment on patients with first-time lateral patella dislocation. Only studies with Level of Evidence 1 or 2 were taken into account

Author	Year	Randomization	Evidence Level	Surgical Procedure	Outcome Parameters	N	Mean Follow-Up Time	Findings	Misc
Apostolovic [3]	2011	no	2	loose body removal/refixation, medial capsular repair, lateral retinacular release	Cincinnati Score, recurrence rate	37	6 years	no difference	
Christiansen [4]	2008	yes	1	MPFL repair	recurrence rate, Kujala Score, KOOS Score	80	2 years	no difference	
Bitar [5]	2012	yes	1	MPFL reconstruction	recurrence rate, Kujala Score	41	44 months	surgery significantly superior in all parameters	
Camanho [6]	2009	yes	2	MPFL repair	recurrence rate, Kujala Score	33	3 years	surgery superior in all parameters	only “statistical trends”
Sillanpää [7]	2008	no	2	MPFL repair	recurrence rate, “return to sports”, Kujala Score	61	7 years	surgery superior in “return to sports”, rest: no difference	
Sillanpää [8]	2009	yes	1	medial repair or Roux-Goldthwait procedure	recurrence rate, “return to sports”, Kujala Score	40	7 years	surgery superior in recurrence rate, rest: no difference	
Petri [9]	2013	yes	1	MPFL repair	recurrence rate, Kujala Score, patient satisfaction	20	2 years	no difference	power 25%

Apostolovic et al. performed a prospective, non-randomized-controlled trial comparing 23 conservatively treated patients with 14 surgically treated patients in the context of primary LPD [3]. The criterion to treat surgically was the presence of (osteo)chondral lesions. Besides loose body removal/refixation, the patients received medial capsular repair and lateral retinacular release. The authors reported no differences between groups regarding recurrence rate or Cincinnati Knee scores, which goes along with the findings of Christiansen et al. [4]. Eighty patients with primary LPD were randomized to either conservative therapy or medial patellofemoral ligament (MPFL) repair. Patients were followed 2 years and demonstrated no significant differences between groups with regard to recurrence rate, KOOS Score, and Kujala Score.

However, there are considerable studies describing benefits of an initial surgical procedure. Bitar et al. conducted a randomized-controlled trial on primary LPD [5]. Thirty-nine patients were randomized to either non-operative treatment (immobilization and physical therapy) or reconstruction of the MPFL. At 2-year follow-up, the MPFL group was superior in terms of Kujala scores and recurrence rates. Camanho et al. also investigated patients with primary LPD [6]. Thirty-three patients were randomly assigned to either conservative treatment (immobilization and physiotherapy) or MPFL repair. The authors reported superior results in the surgical group. Two studies very similar to each other were published by Sillanpää et al. in 2008 and 2009 [7, 8]. The outcome parameters recurrence rates, Kujala scores and “return to sports – rate” were determined in patients with conservative treatment and in those with surgery. While the surgical group was superior in terms of “return-to-sports” there were no significant differences in the other outcome parameters [7]. In contrast, Sillanpää et al. stated in the following randomized-controlled study that patients treated surgically were superior in terms of recurrence rate but not in the other parameters [8]. Finally, a randomized-controlled trial published by Petri et al. should be mentioned [9]. In a multicentric approach, patients with primary LPD were randomized to either conservative treatment or MPFL repair. Unfortunately, the trial was discontinued after 20 patients and did therefore not reach sufficient power.

In synopsis of the abovementioned studies, there is no consensus on whether patients with first time LPD should be treated conservatively or surgically (Table 1). Besides, in most of those studies the surgically group was uniform, meaning that all patients were treated with the same surgical procedure. In addition, previous studies have focused more on the rate of re-dislocation and were not based upon modern and relevant PROM scores. Treatment with the same surgical procedure is regarded as a major limitation as surgical treatment of LPD should be done in a tailored manner, depending on the patient’s anatomic pathologies which lead to LPD [10, 11]:

### MPFL reconstruction

Reconstruction of the medial patellofemoral ligament (MPFL) is a proven technique for LPD [10–12] and today’s established standard treatment. However, some authors have reported a considerable complication rate [13, 14]. Many failures were reported due to inappropriate indications. The latter means performing *isolated* MPFL in patients with coexisting severe osseous pathologies like high-grade trochlear dysplasia or a pathologic tuberositas-tibiae-trochlea-groove distance (TT-TG distance) [15, 16]. Isolated MPFL reconstruction is regarded as inappropriate in patients with: (1) TT-TG distance > 20 mm, (2) femoral anteversion ≥ 40° (n. Waidelich/Strecker), (3) high-grade trochlea dysplasia, (4) severe patella alta, and (5) tibiofemoral valgus > 5°. Moreover, it is striking that most abovementioned studies applied MPFL repair. Only Bitar et al. performed MPFL reconstruction [5]. With accurate indications and surgical technique isolated MPFL reconstruction provides a good outcome in patients with LPD [17, 18].

### Trochleoplasty

When the trochlea is flat or convex (dysplasia Dejour type B, C or D) a “deepening trochleoplasty” should be considered. The aim of the trochleoplasty is to (a) reduce the too prominent anterior bone stock, (b) create better conformity with the patella (concave groove) and a lateral trochlea facet as restraint against the lateralizing quadriceps pull, and (c) to lateralize the groove when there is trochlea asymmetry in order to normalize TT-TG distance [19, 20]. Many authors have reported that trochleoplasty leads to a good clinical outcome in patients’ LPD due to a dysplastic femoral trochlea [12, 19, 21–27].

### Tibial tuberosity transfer

The most popular type of osteotomy in the setting of LPD is certainly the osteotomy and transfer of the tibial tuberosity (TTT). Many articles reported good clinical success for medialising TTT in patients with LPD and high TT-TG values [12, 28–32]. Similarly, good results were found for distalizing TTT in patients with LPD and patella alta [33, 34]. TTT can be tailored to the pathology of the patient by performing combined medialization and distalization.

### Derotational osteotomy

Derotational osteotomies of the femur (externally rotating) provide good results in patients with LPD and associated torsional deformities [35–37]. However, the literature is incongruent on whether rotational osteotomies of the femur should be performed at the proximal or distal aspect [38–41]. In the authors hand’s, derotational osteotomy is carried out at the distal femur.

### Varus osteotomy

Distal femoral varus osteotomies have been described in the recent years to be successful in the management of patellofemoral instability with genu valgum [42]. They have been described as promising and useful in the management of patellofemoral instability with genu valgum, leading to an improved patellar alignment and to a reduction in the risk of recurrent patella dislocation [42, 43].

### Aims of the study

Due to the abovementioned lack of consistent evidence, it is the aim of the study to investigate whether patients with primary LPD are better treated conservatively or operatively (tailored surgery to stabilize the patella).

### Hypotheses

It is hypothesized that patients with primary LPD when treated either conservatively or surgically (tailored stabilizing procedure) will show significant differences *after two years* in terms of the Banff Patellofemoral Instability-Instrument (BPII) 2.0 (hypothesis 1). We assume as well that the abovementioned groups also differ significantly in terms of recurrent patella dislocations (hypothesis 2).

## Methods

### Study design and participants

A prospective, randomized-controlled trial design is applied. Before commencement of the study, approval of the ethical committees (EC) of the participating centers is obtained. Patients with objective, unilateral first time LPD, based on patient’s history and physical examination, will be considered for participation. After written informed consent the patients are included in the study at their first visit at the emergency department. Excluded are (a) patients with osteochondral lesions requiring removal/refixation, (b) patients with recurrent LPD, (c) pregnant patients, and (d) patients > 45 years of age and patients with physical maturity Kramer stage 1 to stage 3a [44, 45].

Clinical workup includes thorough history, physical evaluation, plain radiographs, and magnetic resonance imaging (MRI) in all patients. When the physical examination reveals suspicion of maltorsion syndrome, MRI (or CT scan) is done of the hip, knee, and ankle to quantify femoral and tibial torsion. When the physical examination reveals suspicion of a relevant genu valgum or varum a long-standing x-ray is performed.

### Randomization

Patients successfully included in the study are then randomized to either conservative treatment or to a tailored patella stabilizing treatment. Block randomization will be used to ensure a balanced ratio of group sizes. The total number of patients will be divided into blocks of the same size. This will be followed by a balanced but random allocation of the treatment groups in the individual blocks. Patients will be divided into 10 blocks of 16 patients each and will be assigned to the two different treatment groups.

Block randomization is performed in advance with SPSS (IBM SPSS statistics, Chicago, IL, USA) to guarantee equal group sizes. Syntax and seed for the random number generator are kept for reproducibility of the processes.

### Documentation

The investigator records the participation on a special identification list of patients. This list gives the possibility for a later identification of the patients and contains the patient number, full name, date of birth, and the date of the enrolment into the study. The identification list of patients remains in the study center after the closure of the study. It is the responsibility of the investigator to document all data of the clinical study correctly and completely into the database. Important harms or unintended effects will be recorded in each group.

### Dropouts and exclusion of the clinical trial

To account for 20% attrition during the study period (“dropouts”), it is planned to recruit 80 patients per study group (160 overall) at baseline (see section “Statistics”). If patients request *after* randomization on their own free will a different treatment method — for example, if a patient requests surgery in conservative group — they have to be excluded from the study.

### Interventions

In both groups, in case of relevant hemarthrosis and danger of skin or soft tissue exposure, a joint aspiration is performed [46].

### Conservative group

In the conservative group, patients use a motion-restricting knee brace that (a) protects the patella from lateralization and (b) limits knee range of motion. The range of motion limitation is set to 0-20-40° for week 1-2, 0-10-60° for week 3–4, and 0-0-90° for week 5-8. Partial weight-bearing is applied for week 1 and 2. Patients are prescribed outpatient physical therapy following a protocol suggested earlier by the “Patellofemoral Committee of the German-Speaking Arthroscopy Society (AGA)” [46]:

- *Phase 1 (week 1 + 2)*. Range of motion 0-20-40°, partial weight-bearing
- *Phase 2 (week 3 + 4)*. Range of motion 0-10-60°, progression to full weight-bearing, emphasis on quadriceps recruitment (especially vastus medialis)
- *Phase 3 (week 5–8)*. Range of motion 0-0-90°, re-acquiring activities of daily living, core stability, sensorimotor training (leg axes stabilization), strength training
- *Phase 4*. Return to sports, dependent on the type and previous level of sports activity, gradual increase of training volume and intensity

### Surgical group

The surgical treatment is performed in a tailored manner, addressing the respecting pathologic anatomy that predisposes to LPD. MPFL reconstruction is performed in every patient. Other surgical techniques listed below will be applied in individual combinations, dependent on patient’s needs.

### MPFL reconstruction

#### MPFL reconstruction is applied in all patients of the surgical group

MPFL reconstructionhas been reported with a high variety of surgical techniques (graft type, single vs. double bundle, type of fixation, etc.). The specific surgical technique is carried out on surgeon’s preference at the respective center of the multicentric study.

### Trochleoplasty

Deepening trochleoplasty will be carried out in those patients of the surgical group who suffer from high-grade trochlea dysplasia.

### Tibial tuberosity transfer

Medializing TTT will be applied to those patients with TT-TG distances ≥ 18 mm in the MRI. Distalizing TTT will be applied in patients with Caton-Deschamps Index > 1.2 [47].

### Derotational osteotomy

In those patients with femoral antetorsion ≥ 40° (n. Waidelich/Strecker), a distal femoral derotational osteotomy is carried out. The precise surgical technique for that procedure is given over to the surgeon of the respective center.

### Varus osteotomy

In patients with valgus clinical appearance, a weight-bearing long-standing x-ray should be performed to precisely assess the degree of the deformity in the frontal plane (mechanical femorotibial angle).

In cases with a mechanical femorotibial angle > 5°, a varus osteotomy is performed at the location of the deformity.

Applying a “pragmatic” surgical approach, *not each* single pathology in the patient’s anatomy is addressed. Instead, a maximum of 3 surgical techniques (including the MPFL reconstruction) are performed in one patient. This means, that the 3 leading pathologies are surgically addressed, even in the rare case that 4 or more surgical techniques could be considered. In this sense, lateral release or lateral lengthening and arthroscopy are — for this protocol — not counted as distinct techniques.

## Outcome parameters

### Patient-reported outcome

The Banff Patellofemoral Instability-Instrument (BPII) 2.0 was reported as valid, reliable, and responsive patient-reported outcome tool in the field of patellofemoral instability [48, 49] and is used in the validated German version [50]. The BPII 2.0 serves as one of two major outcome instruments (hypothesis 1).

For exploratory reasons, the following further patient-reported parameters will be assessed in both groups: As second disease specific score, patients accomplish the Kujala Score [51] which was quoted as a reliable, valid, and responsive tool for patellofemoral disorders [52, 53]. In addition, the Short-Form 12 is used (version 2, German; SF-12v2) [54] to determine the general health outcome and the Marx activity scale to rate a patient’s physical activity [55]. The Marx Score asks for the highest activity in the last year. For postoperative monitoring of a patient’s activity, a “modified version of the Marx Score” will be used that refers to the last 2 months.

All abovementioned outcome scores are self-administered and will be assessed preoperatively, 6 and 12 months postoperatively, and then yearly. Those scores are collected during routine visits at the hospital. Alternatively, the questionnaires for assessment of the outcome scores can be filled out online by the patients to reduce dropouts.

The follow-up period is planned for 2 years. However, with the patient’s consent, the period can also be prolonged.

### Other outcome parameters

Recurrence rate is assessed as second major outcome parameter (hypothesis 2). To keep proper medical records on recurrent patella dislocations, the patients are interviewed by telephone on a monthly basis (in addition to the abovementioned visits at the hospital).

The apprehension test is assessed by an experienced observer during the abovementioned routine clinical visits (grade 0, no evasion; grade 1, slight evasion/avoidance; grade 2, gross evasion/avoidance; grade 3, patient too anxious to allow the test).

Joint degeneration is assessed preoperatively and every three years postoperatively by means of MRI (PD-FSE with fat-sat high-resolution in all three planes/T1-TSE, sagittal/T2 weighted, isotrope 3D sequence sagittal reformatted in all three planes). The semi-quantitative MRI Osteoarthritis Knee Score (MOAKS scoring) is applied to rate the degenerative changes determined by MRI [56]. The MOAKS scoring is determined by always the same experienced musculoskeletal radiologist. Additionally, TT-TG distance is measured on postoperative MRI.

In addition, the Patella Instability Severity Score is assessed for exploratory reasons [57].

### Statistics

Patient characteristics will be presented as means, standard deviations, and percentages. The main analysis will use linear mixed models that allow data modeling with a varying number of assessments per patient and time-varying covariates. Such a model will be used to compare the differences in changes over time between the two study groups. The following terms will be included in the model: a random baseline, a first-order autocorrelation covariance matrix, a fixed-effect patient group, a fixed effect time point, and the group-by-time interaction (reflecting the intervention effect). The BPII 2.0 will serve as the primary outcome parameters. The main analysis is “intention to treat” and will focus on the group difference in the change of BPII 2.0 scores between pre-op and 2-year follow-up. The abovementioned secondary outcome parameters will be analyzed with the same model.

Power/sample size analysis was done for the comparison of the two study arms at a 2-year follow-up with regard to the BPII Score. As there are no specific minimal important difference (MID) for the BPII available from the literature, we defined the MID to be 0.5 standard deviations following general recommendations from the literature [58]. A sample size of N = 64 per study arm (128 overall) provides 80% power (alpha = 0.05, two-tailed) to detect a difference of 0.5 standard deviations in a t-test for independent samples. To account for 20% attrition during the study period (“dropouts”), we plan to recruit 80 patients per study group (160 overall) at baseline.

## Data Availability

The datasets used and/or analyzed during the current study are available from the corresponding author on reasonable request.

## Abbreviations

3D: Three-dimensional
AGA: Gesellschaft für Arthroskopie und Gelenkchirurgie
BPII: Banff Patellofemoral Instability-Instrument
CT: Computer tomography
EC: Ethics committee
ICF: Informed consent form
LPD: Lateral patella dislocation
MID: Minimal important difference
MOAK scoring: MRI Osteoarthritis Knee Score
MPFL: Medial patellofemoral ligament
MRI: Magnetic resonance imaging
N: Sample size
PD-FSE: Proton density-fast spin echo
PF: Patellofemoral
PROM: Patient-reported Outcome Measures
SF-12v2: Short-Form 12 version 2
TSE: Turbo spin echo
TTT: Transfer of the tibial tuberosity
TT-TG distance: Tuberositas-tibiae-trochlea-groove distance
